# Multidisciplinary management of pregnancy-associated and early post-partum head and neck cancer patients

**DOI:** 10.3389/fonc.2023.1298439

**Published:** 2023-11-22

**Authors:** Cristiana Bergamini, Stefano Cavalieri, Carlo Resteghini, Salvatore Alfieri, Imperia Nuzzolese, Elena Colombo, Arianna Ottini, Giuseppina Calareso, Andrea Vingiani, Nicola Alessandro Iacovelli, Marzia Franceschini, Marco Guzzo, Alberto Deganello, Lisa Licitra

**Affiliations:** ^1^ Head and Neck Medical Oncology Department, Fondazione IRCCS Istituto Nazionale dei Tumori, Milan, Italy; ^2^ Department of Oncology and Hemato-Oncology, University of Milan, Milan, Italy; ^3^ Radiology Department, Fondazione IRCCS Istituto Nazionale dei Tumori, Milan, Italy; ^4^ Pathology Department, Fondazione IRCCS Istituto Nazionale dei Tumori, Milan, Italy; ^5^ Radiotherapy Department, Fondazione IRCCS Istituto Nazionale dei Tumori, Milan, Italy; ^6^ Head and Neck Surgery Department, Fondazione IRCCS Istituto Nazionale dei Tumori, Milan, Italy

**Keywords:** head and neck cancer, prognosis, multidisciplinary management, pregnancy associated cancer, diagnosis

## Abstract

**Background:**

Pregnancy-associated cancer (PAC) occurs during pregnancy or within 12 months after the delivery. Head and neck cancer (HNC) during pregnancy is infrequent, therefore diagnosis and personalized therapy are intricate.

**Methods:**

We investigated outcomes of 15 PAC patients (5 salivary, 4 nasopharyngeal, 3 thyroid, 2 oral cavity, one HPV-related carcinoma) diagnosed in the period 2005-2019. A literature review on PAC is provided.

**Results:**

Median gestational age at PAC diagnosis was 28 weeks (range: 16–40 weeks) in ten cases, at 5 months after delivery (range: 1 week–6 months) in the remaining five. Treatments included surgery (3 during pregnancy, 5 after childbirth), chemoradiation (8), and 3 patients with upfront metastatic disease received chemotherapy. Median survival was 6.6 years (eight women remain with no evidence of disease six years after diagnosis).

**Conclusion:**

All patients received state-of-the-art therapy, with encouraging long-term results, highlighting treatment safety in women with HNC during pregnancy.

## 1 Introduction

Cancer is one of the most common causes of death in women during their reproductive years (1), though, cancer during pregnancy is an atypical clinical issue (2). Pregnancy-associated cancer (PAC) is usually defined as a malignancy diagnosed during pregnancy and up to 1 year after the end of pregnancy. Current knowledge about PAC is limited because PAC is rare (3), with an estimated incidence of 1 in 1000 pregnancies (4). Maternal age distribution influences PAC incidence rates because the risk of cancer increases with age (5). With the increase in average maternal age over the last 30 years (6) a rising incidence of PAC might be expected.

Due to its rarity, the diagnosis of PAC might be delayed, leading to a potentially more advanced stage of disease at presentation, with subsequent worse outcomes. The most prevalent PACs are breast cancers (1 out of 3000 pregnancies) (7), followed by brain, cervical, gastrointestinal, genitourinary, lymphoma, leukemia, melanoma, and ovarian, cancers (1, 8, 9). Pregnancy-associated head and neck cancers (PA-HNC) among PACs are exceedingly rare, accounting for only 0.4% of all HNC diagnoses in women aged 16–49 years (10).

The management of PA-HNC represents a substantial challenge. Pregnant patients should be treated with the equivalent intensity adopted for non-pregnant individuals. However, treatment selection and the choice of its administration should be adapted to ensure the mother’s and her baby’s safety. In the literature, there is no consensus about the definition of PA-HNC, defined as HNC diagnosed either during pregnancy, lactation, or up to 1 year post-delivery (11, 12).

The most frequently reported PA-HNCs are laryngeal carcinoma, thyroid carcinoma, melanoma and lymphoma (13). Nasopharyngeal carcinoma in pregnant women has been reported in endemic areas, such as Asia, Northern Africa, and among Inuit populations (14–16).

Younger age at HNC diagnosis (17–20), together with the tendency to delay pregnancy until late reproductive age, have increased the risk of PA-HNC (21–26). Although the tumorigenic role of hormones was hypothesized, currently, there is no evidence that pregnancy in itself may increase the risk of HNC (26, 27).

These comparatively infrequent malignancies deter in conducting extensive studies examining their diagnosis, management and outcomes. The present work describes a single institution case series of 15 patients with PA-HNC. The available data on PA-HNC and the consequence of pregnancy on cancer prognosis are summarized. Moreover, we reviewed the medical, surgical, and radiation oncological routes chosen in the care of pregnant patients with HNC. The novelty of this work is the multidisciplinary view of the patients’ management as well as the literature review and the provided recommendations.

## 2 Materials and methods

### 2.1 Patient selection

In this retrospective study, we analyzed data from medical charts of consecutive patients with HNC during pregnancy diagnosed and treated at the Fondazione IRCCS Istituto Nazionale dei Tumori of Milan, Italy, from 2011 to 2020. All cases were discussed in multidisciplinary HNC tumor board meetings, including gynecologists. Socio-demographic (age and comorbidities) and clinical details (gravidity, diagnosis, cancer detection method, symptoms, tumor histology, treatment features, pregnancy and neonatal outcomes, and mother’s vital status) were recorded. Cancers were staged according to the eighth edition of the AJCC/UICC staging systems.

The present study was approved by the Ethical Committee of Fondazione IRCCS Istituto Nazionale dei Tumori, Milan, Italy in November 2020 (local study identifier INT 268/20).

### 2.2 Statistical methods

Patient characteristics were analyzed with descriptive statistics as appropriate. Survival curves were estimated using the Kaplan-Meier method, and median follow-up using the reverse Kaplan-Meier method. Statistical analyses were performed using Prism GraphPad (version 5.02) software.

## 3 Results

### 3.1 Patient characteristics

Fifteen cases were included in this study, with the median age at diagnosis being 37 years (range: 27–43 years). The most frequent tumor sites were salivary glands (five patients; 33%), nasopharyngeal carcinoma (four; 27%), thyroid cancer (three; 20%), oral cavity squamous cell carcinoma (two; 13%), and HPV-related oropharyngeal squamous cell carcinoma (one; 7%). Patient characteristics are described in **Table 1**.

**Table 1:** Patient characteristics of the case series (15 pregnant patients with HNC).

Variables	Number	%
Age (years)		
<25 25-29 30-34 35-39 >40	0 2 3 6 4	0 13 20 40 27
Gravidity		
1 2-3 >3	12 3 0	80 20 0
Education		
Elementary school Middle school High school	0 0 15	0 0 100
Marital status		
Single Married Other	0 14 1	0 94 6
Familial risk of cancer		
Yes Unknown	0 15	0 100

In 10 patients (67%), HNC diagnosis occurred at a median gestational age of 28 weeks (range: 16–40 weeks). The remaining five patients (33%) were diagnosed after a median of 5 months from delivery (range: 1 week–6 months).

In one asymptomatic patient, cancer diagnosis was incidental, whereas the remaining 14 individuals (93%) had HNC-specific symptoms at diagnosis. One-third of patients were diagnosed at an early stage (stage I, 14%; stage II, 20%), and the remaining 66% had loco-regionally advanced disease (stage III, 26%; stage IV, 40%). Radiological assessments were performed after childbirth in 80% of patients. Seven women had nodal involvement and four had distant metastases at diagnosis (**Table 2**).

**Table 2:** Tumor characteristics, clinical profile, characteristics of diagnosis and therapeutic management.

Case	ECOG PS	Tumor site	Histology	Age *(year)*	Pregnancy week	Pregnancy complications	Mode of cancer detection	Stage	M1**	Therapy	OS (years)	Maternal outcome	Fetal outcome
1	1	Salivary glands	Adenocarcinoma	43	22	yes	Symptom	II (T2 N0 M0)	absent	S*; RT	1.22	NED	Vital
2	3	Thyroid	Differentiated carcinoma	38	32	no	Symptom	IV (T3 N1b M1)	present (bone, lung, mediastinal nodes, soft tissue)	S; RT; CT (1L CBDCA + ADM; 2L lenv)	4.63	DOD	Vital (cesarean section)
3	0	Salivary glands	Salivary duct carcinoma	27	28	no	Symptom	IV (T3 N2b M1)	present (lung)	RT; CT (CDDP + trastuzumab)	3.00	DOD	Vital
4	0	Oral cavity	Squamous cell carcinoma	27	26	no	Symptom	I (T1 N0 M0)	absent	S*	1.58	DOD	Vital
5	0	Salivary glands	Mucoepidermoid Carcinoma	32	28	no	Symptom	II (T2 N0 M0)	absent	S*; RT	5.72	DOD	Vital
6	0	Nasopharynx	Undifferentiated carcinoma	37	>40	yes	Symptom	IV (T4 N2 M0)	absent	RT	9.52	NED	Vital
7	0	Nasopharynx	Undifferentiated carcinoma	30	>40	yes	Symptom	III (NA)	absent	RT	12.42	NED	Vital
8	0	Salivary glands	Adenoid cystic carcinoma	36	28	no	Symptom	IV (T4a N0 M0)	absent	S	10.76	DOD	Vital
9	0	Nasopharynx	Undifferentiated carcinoma	37	16	no	Symptom	III (T2 N1 M0)	absent	RT	5.74	NED	VIP
10	1	Salivary glands	Myoepithelial carcinoma	41	>40	no	Symptom	IV (NA)	present (lung)	RT; CT (ADM)	10.78	DOD	Vital
11	0	Thyroid	Medullary carcinoma	37	18	no	Symptom	II (T2 N0 M0)	absent	S; RT	7.55	DOD	Vital
12	0	Nasopharynx	Undifferentiated carcinoma	34	28	no	Symptom	III (T1 N2 M0)	absent	RT	6.43	NED	Vital
13	1	Thyroid	Differentiated carcinoma	37	40	yes	Fortuitous	IV (T2 N0 M1)	present (bone)	S; RT	10.67	ED	Vital (cesarean section)
14	0	Oral cavity	Squamous cell carcinoma	40	>40	no	Symptom	I (T1 N0 M0)	absent	S; RT	4.82	NED	Vital
15	0	Oropharynx	Squamous cell carcinoma	40	>40	no	Symptom	III (T2 N1 M0)	absent	RT	3.27	NED	Vital

1L, first-line; 2L, second-line; ADM, doxorubicin; CBDCA, carboplatin; CDDP, cisplatin; DOD, died of disease; ECOG PS, Eastern Cooperative Oncology Group Perfomance Status; lenv, lenvatinib; NA, not available; NED, no evidence of disease; S, surgery; RT, radiotherapy; CT, chemotherapy; VIP, voluntary interruption of pregnancy; *Treatment during pregnancy; ** M1 staging was assessed after birth in all cases.

### 3.2 HNC management

Three women (cases 1, 4 and 5 in **Table 2**) received cancer treatment during pregnancy. Surgery of the primary tumor was performed in three patients (two with salivary gland cancer and one with oral cavity cancer). After childbirth, five patients received surgery (three thyroid cancers - of which two with metastatic disease, one patient with salivary gland cancer, and one with oral cavity cancer), eight patients received concomitant chemoradiation with curative (33%) or postoperative intent, and three of four patients with metastatic disease at diagnosis were treated with chemotherapy (**Table 2**).

### 3.3 Maternal and pregnancy outcomes

Delivery occurred at term in 73% of individuals. In the remaining patients, the pregnancy outcome was elective childbirth in 3 patients, and a voluntary abortion related to PAC diagnosis was induced in one case. Cesarean section was chosen instead of vaginal delivery in two metastatic thyroid cancer patients due to their disease (pelvic bone metastases in one case; bulky mediastinal tumor involvement with dyspnea and bone metastasis-related lumbar pain in the second woman).

All 14 live births were reported with satisfactory neonatal conditions (APGAR not available). One premature childbirth was induced at 30 weeks due to growth retardation during pregnancy. Good neonatal conditions were reported for all 14 live births, with neither congenital anomalies nor major maternal morbidities.

In the PA-HNC cohort, the median overall survival was 5.8 years (range: 14+ months–12+ years). Eight patients still remain in complete remission six years after diagnosis.

Seven maternal deaths occurred during the study: three patients were affected by salivary gland cancer, two patients by thyroid cancer, one by oral cavity cancer, and one patient with salivary gland cancer died due to SARS-CoV-2 infection. Out of the 8 survivors, at last follow-up 7 were alive and cancer-free, while only one was alive with evidence of disease (**Table 2**). Median follow-up was 114.41 months (95% CI 57.96-NR), median overall survival was 129.31 months (95% CI 55.62-NR).

The median time between diagnosis and death was 5.5 years (range: 19 months–10 years). In one case, childbirth occurred at 30 weeks of pregnancy. A fetus up to 28 weeks is deemed as ‘severe preterm’ when the chances of neonatal death or permanent disability are high. In this scenario, delivery after 37 weeks is recommended without compromising the mother’s safety.

## 4 Clinical features of the case series and literature review

In the following paragraphs, while presenting the management of the selected PA-HNC patients, we reviewed the relevant literature on the topic, referring to the current procedure that would have been offered to non-pregnant individuals. Pregnancy termination should not be justified by the cancer diagnosis itself. Treatment was similar to that for non-pregnant patients, except that radiation is not recommended at any stage of pregnancy, and the choice of delivering chemotherapy should be cautiously evaluated case by case. For ideal clinical decision-making, a multidisciplinary approach is mandatory.

### 4.1 Clinical presentation

The vast majority of our patient population (14 cases) was diagnosed with HNC in a symptomatic phase. Most HNCs were diagnosed as self-palpated cervical masses or painful ulcerated mucosal lesions.

Older maternal age is associated with mutation accumulation, and this might lead to an increased risk of malignancies. Recent studies reported that epithelia from these tumors contain a high expression of essential hormone-regulated genes linked to cell proliferation, metabolism, tumor aggressiveness and recurrence. Breast cancer is one of the most extensively studied malignancies during pregnancy. Notably, breast cancer cells have a significantly higher expression of genes guiding the cell cycle process, most of which are hormone-dependent (28).

One thyroid cancer patient had venous thromboembolism (VTE) and required blood transfusions. However, these complications are expected in pregnancy-associated thyroid cancers (29). The babies delivered by the three thyroid cancer patients under study had no harmful neonatal outcomes, no inborn malformations, intrauterine growth limitation, fetal death or premature labor.

### 4.2 Imaging and staging procedures

The radiological staging was performed after childbirth in 80% of our patients. Most studies and reviews considered PAC to have a suboptimal prognosis due to a late diagnosis and the restrictions in oncologic therapy. Any treatment delay may impair the maternal prognosis. Therefore, radiological staging should be offered when malignancy is suspected or known.

In all our patients, ultrasonography (US) was the first line imaging technique evaluating the cervical mass during pregnancy because of the ability of US to differentiate between solid and cystic lesions with sufficient sensitivity (30). Besides, the US lacks ionizing radiation, negating the possible cause of congenital disorders.

Theoretically, computed tomography (CT) could be performed during any trimester of pregnancy with proper abdominopelvic shielding. When the fetus is outside the field of view, the radiation exposure is estimated to be low for CT. The mean fetal dose produced from a chest CT is 0.06 mGy with a maximum possible limit of 0.96 mGy — less than half an amount than an abdominal radiograph (31). However, in daily operations, CT should be avoided because the internal scatter of the radiation to the fetus cannot be evaded (32).

The best radiologic assessment for HNC is Magnetic Resonance Imaging (MRI); however, contrast-enhanced MRI scans are not recommended during pregnancy (33). Gadolinium‐based contrast, with gadolinium as a potential teratogen, can cross the blood‐placental barrier (34). During breastfeeding, contrast-enhanced MRI scans are considered safe (35). The images may be difficult to be interpreted due to the increased background enhancement related to hypervascularization and inflammatory changes. Although negligible doses of gadolinium‐based agents are known to be excreted into breast milk, the chances of complications, such as direct toxicity or allergic reactions, are minimal and have not been reported. Weighing the nominal risks, the American College of Radiology endorses the safety of breastfeeding after MRI. However, avoidance of breastfeeding for the 12-24 hour period after gadolinium administration is recommended (36). Although accurate MRI scans should be obtained after contrast administration, functional imaging techniques, such as Diffusion Weighted Imaging (DWI), may help obtain a satisfactory tumor delineation independently of contrast administration.

Systemic staging studies are indicated for advanced cancers. Nevertheless, pregnant and non-pregnant patients are differently managed due to radiation risks and the deleterious effects of the contrast agents on the developing fetus or embryo. In pregnant patients, positron emission tomography/CT (PET/CT) exposes the fetus to a comparatively high radiation dose caused by the 18F-FDG uptake and CT dose combination. Therefore, PET/CT is recommended to be deferred until after the pregnancy completion (37).

In addition, placental histology is monitored in women with malignant melanoma or metastatic disease to evaluate the fetal risk of metastasis (38, 39). Even though we lack this information in our patient cohort, the application of non-invasive prenatal testing, equipped to detect preclinical cancer, might lead to an earlier cancer diagnosis at a preclinical stage (40).

### 4.3 Surgery

In our case series, five patients were treated with surgery after childbirth. Two patients were affected by initial HNC (respectively by papillary thyroid carcinoma and oral squamous cell carcinoma). Conversely, three patients had advanced disease (two patients with metastatic thyroid carcinoma, respectively with papillary and medullary carcinoma, and one with locally advanced adenoid cystic carcinoma). While these three patients showed an advanced HNC, the maternal treatment was not delayed because all patients received state-of-the-art surgical treatment as per their clinical condition. Surgery was safely performed without any delay.

For all patients with gestational thyroid cancer needing surgery, a team of proficient endocrinologists and oncologists is fundamental to personalize their treatment.

Even though neck lymph node dissection is fundamental in the proper management of locally advanced HNC amenable to surgery, our patients were only operated on primary tumors. This has been planned due to HNC histology (adenocarcinoma and mucoepidermoid carcinoma for salivary gland carcinomas) or the limited stage at diagnosis (for oral squamous cell carcinoma).

Surgical recommendations for PA-HNC patients are comparable to those for patients who are not pregnant. Indications depend on the clinical stage, genetic status, tumor biology, prenatal age, and the mother’s surgical requests. Gestational age at diagnosis is vital for surgical planning because of the associated risks of spontaneous abortion and preterm labor (21). For this reason, the HNC multidisciplinary team should also involve maternal-fetal medicine specialists.

Surgery can be safely executed anytime during pregnancy providing maternal and embryo/fetal safety is addressed. Pregnancy-induced changes in maternal physiology and anatomy majorly impact surgical planning because they may determine potential issues for the mother and fetus/embryo receiving anesthesia. The baby may be exposed to potential danger due to intraoperative hypoxemia or asphyxia triggered by several physiological alterations within the maternal and fetal bodies. Additionally, exposure to teratogenic drugs and the risk of premature delivery because of the surgical process or administered drugs are equally harmful (41). Adverse post-surgery fetal outcomes, suggested by a population study, may be triggered by the underlying maternal disease instead of the direct influence of anesthesia (42). In most cases, the fetus passively receives the anesthesia from the mother, does not bear blood losses and experiences passive changes but direct alterations instigated by surgeries. Despite the minimal teratogenicity of anesthetic agents, surgery is not usually advised until after the first trimester to lessen possible risks to the fetus.

The current guidelines propose to avoid optional surgeries until the second or third trimester (43), with the second trimester being the safest period. The stress of surgery can lead to premature labor and premature delivery during the third trimester. With access to a neonatal intensive care unit, neonatologic and obstetric help should be in place (43, 44).

### 4.4 Radiation therapy

In this case series, eight PA-HNC patients were treated with radiation therapy after childbirth, five patients with radical intent, and three cases in the postoperative setting.

The safe administration of radiation to pregnant women is challenging. The highest sensitivity of fetal cells to radiation occurs during early organogenesis (up to the eighth gestational week), with doses over 0.05 Gy. The most common radiation-induced abnormality in this setting includes developmental disability. In PA-HNC patients diagnosed during the first trimester, treatment with radiation (with fetal exposure >0.1–0.2 Gy) is linked to a greater risk of congenital abnormalities. Therefore, these cases must be recommended for pregnancy termination (45).

Radiation therapy is generally not offered to PA-HNC patients because of the following significant risks: teratogenicity, probable installation of childhood malignancies and hematological disorders. The fetal developmental stage and the dose, intensity, and distribution of radiation are directly connected to irradiation toxicity during pregnancy. During the first trimester, radiation-induced growth and mental deficiency may take place (46).

The radiotherapy-related risks can be lowered by avoiding the direct exposure of the fetus to radiation by utilizing pelvic shielding or modifying the beams’ physical characteristics limiting the dose delivered to the fetus. However, these therapeutic adjustments may lead to suboptimal management of PAC (47–49).

Nonetheless, several successful radiation treatments during pregnancy, with the birth of healthy children, have been described (50–57).

Luis et al. reported that out of the 109 cases following up on the offspring, 13 reported adverse outcomes, including spontaneous abortions, perinatal deaths and neurological deficits (58). It is emphasized that if radiotherapy is required before the post-partum period, treatment should be managed by a qualified team of physicists and radiation oncologists, ensuring careful planning, appropriate shielding devices, and distribution of fractional doses for a prolonged period reducing the scattered dose to the fetus. In this setting, there is evidence that an accurate pre-treatment simulation in a PA-HNC patient is fundamental to predicting the fetal dose (59).

However, due to the complexity of treatment (the physicists calculate the fetal radiation dose and adjust the treatment plan), the current European guidelines prefer to delay radiation therapy to the post-partum period, regardless of the treated site, except a site located adequately far from the uterus demands an urgent intervention (60).

### 4.5 Systemic therapy

While considering systemic treatments for PA-HNC patients, pregnancy‐related changes in maternal physiology and fetal developmental stage should be taken into account. These include altered metabolism and clearance that may influence drug bioavailability and toxicity profiles. None of the PA-HNC patients in our case series was treated with chemotherapy during pregnancy.

One of the most relevant factors in choosing and scheduling systemic therapy is the potential consequence of chemotherapy on fetal development. Following implantation (circa two weeks after conception), organogenesis occurs over the subsequent 8-10 weeks. This period has the highest probability of significant malformations and fetal loss (61). Although studies reporting chemotherapy in the first trimester are few, some evidenced fetal abnormalities, including neural tube defects, cardiac defects, cleft lip/palate, and fetal loss (61–63). Therefore, chemotherapy administered during the first trimester, especially during organogenesis (weeks 4 to 12), could potentially result in teratogenesis (3, 64).

Although chemotherapy is contraindicated during the first trimester, cytotoxic chemotherapy is more widely accepted during the second and third trimesters of pregnancy. Low rates (3%–5%) of fetal malformations were reported by most of the studies inspecting chemotherapy safety beyond the first trimester (65–70).

Chemotherapy-associated congenital deformities have been reported at 16%, 8%, and 6% of cases in the first, second and third trimesters, respectively. These fetal consequences in the second and third trimesters include restraint intrauterine growth, prematurity, and lower birth weight. Chemotherapy-induced maternal toxicity may also lead to fetal hair loss and myelosuppression (64).

Chemotherapy regimens used to treat PAC patients include 5‐fluourouracil, doxorubicin, cyclophosphamide (65) and carboplatin plus paclitaxel (71–73). Data in the field are limited, and although taxane and/or platinum therapy bring about encouraging fetal outcomes, these are based on a relatively small number of patients with limited follow‐up.

Taxanes and platinum agents should be employed carefully to treat pregnant patients with salivary gland carcinoma, only if standard anthracycline‐based therapy is not feasible as the preferred option. Another relevant aspect is chemotherapy pharmacokinetics. Indeed, a higher cytochrome P‐3A4 activity is detected in the third trimester. Therefore, a greater taxane clearance is likely, with possible limitations on drug activity (74).

Carboplatin may be preferred over cisplatin due to its better pregnancy-related safety profile. Single-agent platinum regimens have already been reported in this context. Mir et al. evaluated 43 patients with PAC, of whom 28 had ovarian cancer. Cisplatin was found to be linked with various adverse consequences: restricted intrauterine growth (in 8.3% of patients), premature birth (8.3%), respiratory distress (8%), and neonatal anemia (5.6%). Compared with cisplatin, carboplatin does not lead to fetal defects, toxicities, or adverse outcomes in the newborn (73).

For PAC patients treated with chemotherapy during pregnancy, delivery timing must be synchronized with therapies to avoid cytopenias at delivery. Platelets might be transfused, if needed, >30,000/mL for a vaginal delivery or 50,000/mL for a cesarean section. A vaginal delivery is recommended, and a cesarean section should be deemed only for a pelvic tumor (e.g., cervical, anal, or rectal cancer) or routine obstetric symptoms (75).

Thus, single-agent chemotherapy opens up a promising future in managing pregnancy-associated cancers, with a subsided exposure of chemotherapeutic agents to the fetus.

Studies evaluating children exposed to long-term *in utero* chemotherapy imply that chemotherapy is not necessarily linked to inadequate postnatal growth or compromised cognitive or cardiac functions. Nevertheless, more data on long-term outcomes are needed to assess the safety and cancer risk (38).

In the field of ancillary therapy, based on animal and human studies, the effects of corticosteroids are contradictory but tend to designate increased risks in the first trimester. Chemotherapy is feasible after the 14^th^ gestational week, but a few broadly used drugs, like platinum derivatives, taxanes, and etoposide, present substantial infusion reaction events (76). Steroid-based premedications are usually administered to prevent such reactions (77). Corticosteroids are particularly beneficial in these cases. Moreover, the H2 histamine antagonists ranitidine, famotidine and cimetidine are not associated with an increased risk of congenital disabilities (78, 79).

In the field of targeted therapy, no robust data are available. A drug’s placental passage is subjected to its class and size: large molecules, like monoclonal antibodies (e.g., trastuzumab, rituximab), need an active passage through the placenta, which is fully developed at the beginning of the 14th gestational week. On the other hand, tyrosine kinase inhibitors and other small molecules can cross the placenta throughout the pregnancy. Cases of detectable concentrations of antitumor TKI (alectinib in a pregnant woman affected by an ALK-rearranged non-small cell lung cancer) were described, with a fetal plasma concentration at birth 14 times lower than the one observed in the mother (80).

Targeted therapies may increase the risk of fatal morbidity and pregnancy-related difficulties due to the activity of antitumor drugs on biological pathways involved in both tumor pathogenesis and physiologic fetal development.

As angiogenesis is crucial for the placenta’s and fetus’s normal development, the teratogenic angiogenesis inhibitors could incite pregnancy loss, skeletal retardations and fetal growth restriction. Therefore, during pregnancy, anti-vascular endothelial growth factors and other antiangiogenic drugs are avoided (38). One metastatic differentiated thyroid patient included in our case series was treated with lenvatinib after childbirth. Targeted therapies for cancer treatment are not recommended during pregnancy and should be administered after delivery, apart from the possibility of giving rituximab and imatinib in the second and third trimesters (81).

In the framework of immunotherapy, programmed death-1 (PD-1)/PD-L1 and cytotoxic T-cell lymphocyte-4 (CTLA-4) interactions play key roles in maintaining normal fetal tolerance. Nivolumab and pembrolizumab are monoclonal antibodies directed against PD-1. Recently introduced as a cancer therapy agent, anti-PD-1 is considered safe during pregnancy. PD-1 acts in the negative immune regulation crucial for maternal tolerance of pregnancy with an apparent effect on human pregnancy (82). Evidently, immune checkpoint inhibitors like anti-PD1/PD-L1 drugs are associated with increased spontaneous abortion rates in animals (83). In humans, a case of advanced melanoma patient treated with nivolumab was reported during the first seven weeks of pregnancy. Conceivably the first case of a fetal immune-related adverse effect from maternal anti-PD-1 exposure, the prematurely born fetus was identified with intrauterine growth restriction and congenital hypothyroidism (84). Nonetheless, a few case reports identified no miscarriages in melanoma patients treated during their first trimester (85, 86).

To precisely schedule systemic therapy during pregnancy, several factors must be respected: clinicopathologic characteristics (i.e., stage at diagnosis, grade, lymph node and receptor status), the gestational age at HNC diagnosis and the prospect of a full‐term delivery to ensure maternal and fetal outcomes. Based on available data, we would endorse initiating systemic chemotherapy after completing the first trimester without an urgent contraindication. Finally, although milk production may be negatively affected by cancer treatments (87), breastfeedings should be avoided while continuing systemic treatments after birth (88).

## 5 Discussion

Pregnancies complicated by cancer are comparatively rare. However, since women in Western societies tend to delay childbearing until their 30s and 40s, this possibility may be more frequent in the future. In this setting, it is expected that older women may have a higher probability of HNC risk factor exposure (e.g., HPV infection, smoking, alcohol).

Cancer diagnosis during pregnancy is a tricky issue. On the one hand, the mother should be optimally treated, and on the other, the consequences of cancer treatment on the fetus should be minimal (89).

The small number of patients may be a limitation. Other drawbacks of this work are its retrospective nature, the lack of data on patients’ education, and the fact that the study patients were affected by different cancer sites, histologic types, and stages of disease, making it difficult to assess survival outcomes. Nevertheless, the presented data are worthwhile because our case series is a representative sample of PA-HNC treated at a tertiary cancer center.

No major delays between cancer diagnosis and treatment start and no adverse events because of pregnancy were observed in the study cohort.

Given the prevalence of symptoms and the disease stage at clinical presentation in the presented series (all the patients were aware of their pregnant status before the diagnosis), the diagnosis was late for the majority of cases. Literature data reported a higher age-adjusted incidence rate of late stage HNC in men when compared to women in the US (90). In cancer registries we lack data about PA-HNCs, so no direct comparisons can be made between our data and the available literature. However, since almost all cases described here were diagnosed at a late stage, we cannot exclude that pregnancy could have had a promoting action in cancer development and progression. It is well known these phenomena are promoted by complex biological mechanisms. At the same time, pregnancy-related exposures impact fetal growth cell division and organ functioning. The balance between the need to tackle tumor cell proliferation while not impairing normal fetal development is a key point for the principles of PAC management. Indeed, cancer and its treatments are expected to interfere with the complex phenomena of pregnancy.

Cancer diagnosis, staging and treatment are based on the knowledge developed treating non-pregnant HNC patients. To administer the safest and optimal treatment plan to the mother and developing fetus, several challenges in systemic treatments, surgery, radiotherapy and obstetrics must be thoughtfully evaluated in patients with PA-HNC. Indeed, a careful and comprehensive multidisciplinary discussion should be conducted in each case. Given the cited literature, the following factors should be taken into account: maternal age; pregnancy stage; cancer type, site, size and stage; potential embryo-fetal risks associated with anticancer treatment; wishes of the woman and her family; close monitoring of both mother’s and baby’s health during the whole treatment period and in the subsequent follow-up; psychological support.

Cancer treatment delay until achieving fetal maturity may be considered in selected cases, provided that tumor evolution is closely monitored.

The delivery term depends on the date of cancer diagnosis (beyond 35 weeks of gestation in most cases). Pregnancy in itself does not have a deleterious effect on cancer prognosis, but it is often associated with a diagnostic delay.

According to the available evidence, non-obstetrical surgeries may be conducted during pregnancy without any increased risk of adverse outcomes. However, some cancer treatments should be postponed to the second and third trimesters due to the higher risk of fetal harm during the first three months of pregnancy.

## 6 Conclusions

Head and neck cancers during pregnancy present significant ethical and professional challenges for patients and physicians. Several aspects from diagnostic, medical, surgical and radiation oncology standpoints must be addressed to ensure the safety of the mother and the infant. An informed discussion between the patient and her medical team is essential to ascertain a precisely individualized treatment plan maximizing benefits and minimizing risks to the mother and the fetus. Long term effects on children, adolescents and adults, related to maternal cancer treatment during pregnancy should be investigated and longitudinally surveilled.

## Data availability statement

The original contributions presented in the study are included in the article/supplementary material, further inquiries can be directed to the corresponding author: stefano.cavalieri@istitutotumori.mi.it.

## Ethics statement

The studies involving humans were approved by Ethical Committee of the Fondazione IRCCS Istituto Nazionale dei Tumori, via Venezian 1, 20133 Milan, Italy. The studies were conducted in accordance with the local legislation and institutional requirements. The participants provided their written informed consent to participate in this study.

## Author contributions

CB: Writing – original draft, Writing – review & editing. SC: Writing – original draft, Writing – review & editing. CR: Writing – review & editing. SA: Writing – review & editing. IN: Writing – review & editing. EC: Writing – review & editing. AO: Writing – review & editing. GC: Writing – review & editing. AV: Writing – review & editing. NI: Writing – review & editing. MF: Writing – review & editing. MG: Writing – review & editing. AD: Writing – review & editing. LL: Writing – review & editing.
